# Daily irradiation versus irradiation at two- to three-day intervals in stereotactic radiotherapy for patients with 1-5 brain metastases: study protocol for a multicenter open-label randomized phase II trial

**DOI:** 10.1186/s12885-022-10371-3

**Published:** 2022-12-05

**Authors:** Natsuo Tomita, Hiromichi Ishiyama, Chiyoko Makita, Yukihiko Ohshima, Aiko Nagai, Fumiya Baba, Mayu Kuno, Shinya Otsuka, Takuhito Kondo, Chikao Sugie, Tatsuya Kawai, Taiki Takaoka, Dai Okazaki, Akira Torii, Masanari Niwa, Nozomi Kita, Seiya Takano, Shogo Kawakami, Masayuki Matsuo, Tomoyasu Kumano, Makoto Ito, Sou Adachi, Souichiro Abe, Takayuki Murao, Akio Hiwatashi

**Affiliations:** 1 Department of Radiation Oncology, Nagoya City University Hospital, 1 Kawasumi, Mizuho-cho, Mizuho-ku, Nagoya, Aichi 467-8601 Japan; 2 Department of Radiation Oncology, Kitasato University School of Medicine, 1-15-1 Kitasato, Sagamihara, Kanagawa 252-0329 Japan; 3 Department of Radiation Oncology, Gifu University Hospital, 1-1 Yanagido, Gifu, 501-1194 Japan; 4 Department of Radiology, Aichi Medical University, 1-1 Yazako-Karimata, Nagakute, Aichi 480-1195 Japan; 5 Department of Radiation Oncology, Nagoya City University East Medical Center, 1-2-23 Wakamizu, Chikusa-ku, Nagoya, Aichi 464-8547 Japan; 6 Department of Radiation Oncology, Nagoya City University West Medical Center, 1-1-1 Hirate-cho, Kita-ku, Nagoya, Aichi 462-8508 Japan; 7 Department of Radiation Oncology, Ichinomiya Municipal Hospital, 2-2-22 Bunkyo, Ichinomiya, Aichi 491-8558 Japan; 8 Department of Radiation Oncology, Okazaki City Hospital, 3-1 Goshoai, Koryuji-cho, Okazaki, Aichi 444-8553 Japan; 9 Department of Radiation Oncology, Nagoya Ekisaikai Hospital, 4-66 Syonen-cho, Nakagawa-ku, Nagoya, Aichi 454-8502 Japan; 10 Department of Radiation Oncology, Japanese Red Cross Aichi Medical Center Nagoya Daini Hospital, 2-9 Myoken-cho, Showa-ku, Nagoya, Aichi 466-8650 Japan

**Keywords:** Brain metastases, Radiation biology, Stereotactic radiation, Treatment outcome, Clinical Trial, Phase II

## Abstract

**Background:**

Radiobiological daily changes within tumors are considered to be quite different between stereotactic radiotherapy (SRT) (e.g., 50 Gy in 4 fractions) and conventional radiotherapy (e.g., 60 Gy in 30 fractions). We aim to assess the optimal interval of irradiation in SRT and compare outcomes of daily irradiation with irradiation at two- to three-day intervals in SRT for patients with one to five brain metastases (BM).

**Methods:**

This study is conducted as a multicenter open-label randomized phase II trial. Patients aged 20 or older with one to five BM, less than 3.0 cm diameter, and Karnofsky Performance Status ≥70 are eligible. A total of 70 eligible patients will be enrolled. After stratifying by the number of BMs (1, 2 vs. 3–5) and diameter of the largest tumor (< 2 cm vs. ≥ 2 cm), we randomly assigned patients (1:1) to receive daily irradiation (Arm 1), or irradiation at two- to three-day intervals (Arm 2). Both arms are performed with total dose of 27-30 Gy in 3 fractions. The primary endpoint is an intracranial local control rate, defined as intracranial local control at initially treated sites. We use a randomized phase II screening design with a two-sided α of 0∙20. The phase II trial is positive with *p* < 0.20. All analyses are intention to treat. This study is registered with the UMIN-clinical trials registry, number UMIN000048728.

**Discussion:**

This study will provide an assessment of the impact of SRT interval on local control, survival, and toxicity for patients with 1–5 BM. The trial is ongoing and is recruiting now.

**Trial registration:**

UMIN000048728. Date of registration: August 23, 2022.

https://center6.umin.ac.jp/cgi-bin/ctr/ctr_view_reg.cgi?recptno=R000055515.

## Background

**Fig. 1:**
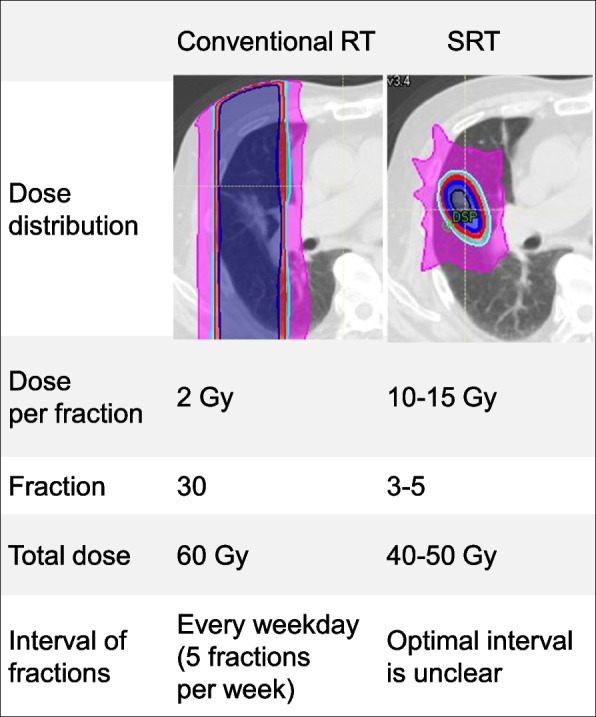
Comparison of practical difference between conventional radiotherapy (RT) and stereotactic radiotherapy (SRT) (e.g., early-stage lung cancer)

Stereotactic radiotherapy (SRT) has improved clinical outcomes for several types of cancers or metastatic lesions [1–4]. A recent trial suggests that when all sites of disease are treated with SRT in patients with oligometastatic disease, this therapeutic strategy can improve overall survival (OS) [5]. SRT is characterized by the use of hypofractionation and much higher doses per fraction (e.g., 50 Gy in 4 fractions) than conventional radiotherapy (RT), which is performed every weekday with multiple fractions using low doses per fraction (e.g., 60 Gy in 30 fractions). Figure 1 shows the concise description for the practical difference between the two methods.

Most tumors have hypoxic tumor cells which are radioresistant [6] and can cause a recurrence after RT [7, 8]. During the period of daily irradiation, surviving hypoxic cells within tumor reoxygenate and get more sensitive to successive irradiation [9, 10]. We previously investigated the variations in the percentage of hypoxic cells after single high-dose irradiation in the murine tumors [11]. As the decrease of the hypoxic fractions continued at least 72 hours after single high-dose irradiation, we consider that reoxygenation continues more than 3 days after single high-dose irradiation. Thus, when SRT is performed on a daily basis, the benefit of reoxygenation can be reduced owing to the use of high-dose irradiation at insufficient intervals. We think that longer intervals more than 24-hour may be necessary to allow more reoxygenation to occur and enhance therapeutic efficacy of SRT.

In this multicenter open-label randomized phase II trial, we aim to assess the optimal interval of SRT and compare outcomes of daily irradiation with irradiation at two- to three-day intervals for patients with one to five brain metastases (BM).

## Methods/design

Participating institutions will be six academic and four general hospitals in Japan. In each participating institution, the institutional review board (IRB) will approve the protocol before patient enrollment occurred. The study was started in September 2022, and participant enrollment will be between September 2022 and March 2026. This trial has been registered with the UMIN-clinical trials registry (UMIN-CTR: UMIN000048728, Date of registration: August 23, 2022, Version 1.0).

### Primary endpoint

Intracranial local control rate (IC-LC), defined as intracranial local control at initially treated sites.

### Secondary endpoints

1. Intracranial progression-free survival (IC-PFS), defined as intracranial PFS at initially treated and at new sites.
2. OS, defined as the time from the date of randomization to death from any cause.
3. Toxicity, assessed by the National Cancer Institute Common Terminology Criteria for Adverse Events (CTCAE) version 5.0.
4. Non-worsening of Karnofsky performance status (KPS), defined as the time from randomization to decline of KPS from any cause.
5. Non-worsening of mini mental status examination (MMSE), defined as the time from randomization to decline of MMSE from any cause.

### Study design

**Fig. 2:**
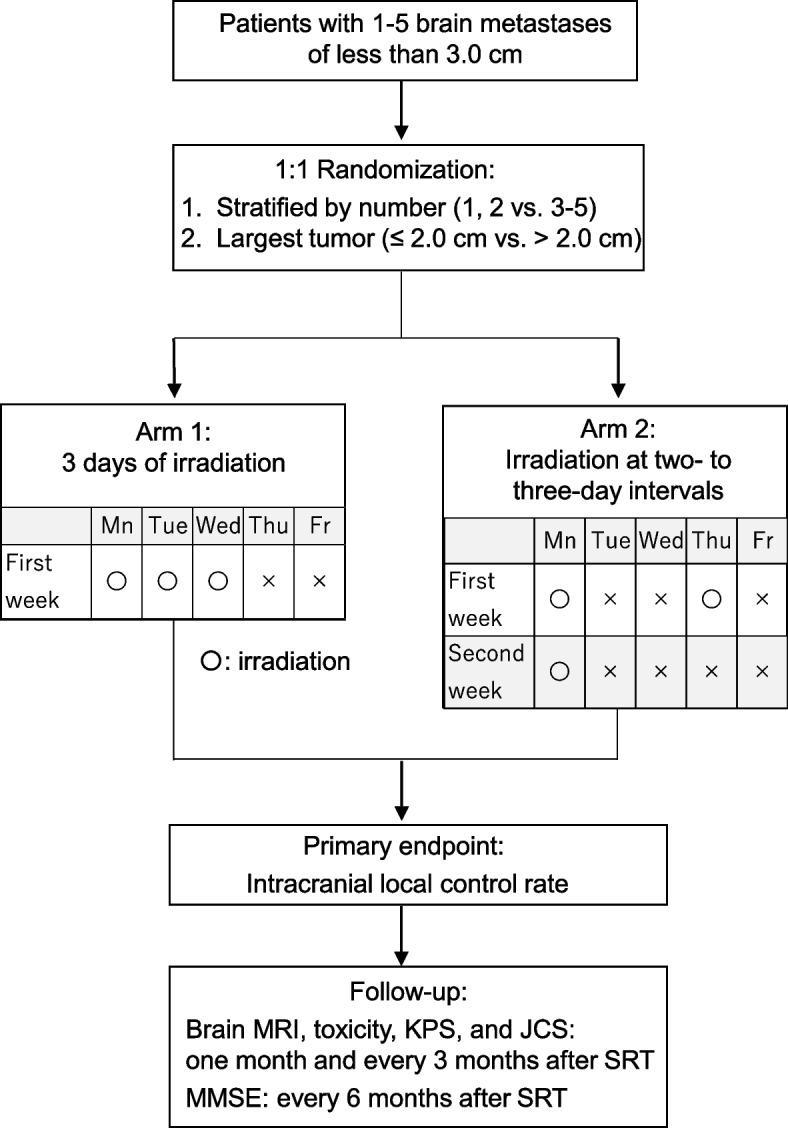
Study schema. MRI: magnetic resonance imaging; KPS: Karnofsky performance status; MMSE: mini mental status examination; JCS: Japan Coma Scale

The study is conducted as a multicenter open-label randomized control phase II trial. Patients will be randomized in a 1:1 ratio between consecutive irradiation (Arm 1) vs. irradiation at intervals (Arm 2) to BM. Patients will be stratified by (1) maximum diameter (≤ 2 cm vs. > 2 cm) among their BM and (2) number of BM (1, 2 vs. 3-5). Figure 2 shows the study schema.

### Inclusion criteria

1. Age 20 years or older.
2. KPS ≥ 70.
3. Total number of 1-5 BM by contrast-enhanced magnetic resonance imaging (MRI).
4. BM with less than 3.0 cm diameter by contrast-enhanced MRI.
5. Willing to provide informed consent.

### Exclusion criteria

1. Histological type of the primary site is lymphoma, small cell lung cancer, and germ cell tumor.
2. Metastases of the brainstem.
3. Meningeal carcinomatosis.
4. History of whole brain radiation therapy (WBRT) or substantial overlap with a previously treated radiation volume.
5. Surgical history for BM.
6. Difficult to be enrolled to the study by reason of insanity.
7. Inability to use enhancing agent for MRI due to low renal function or allergy.
8. In pregnancy or with expectation of pregnancy.
9. Physician dismiss as subject of the study.

### Pre-treatment evaluation

Re-staging is performed within 8 weeks prior to randomization. ^18^F-fluorodeoxyglucose (18-FDG) positron emission tomography (PET) -computed tomography (CT) or a contrast-enhanced CT of the neck to the pelvis is recommended for the evaluation of extracranial metastases. In cases where a contrast agent is unavailable due to impaired renal function or allergic intolerance, a plain CT is allowed. Before random assignment within 2 weeks, all patients are necessary to be evaluated by an MRI of the brain. MRI is performed with a gadolinium-enhanced T1 3D Fast Field Echo and 3D reconstruction of the axial, sagittal, and coronal planes with 1 mm slice thickness (thin-slice MRI). In addition, each patient undergoes a baseline evaluation consisted of medical history including prior treatments of cancer and concomitant anticancer medications, symptom burden, KPS, Japan Coma Scale (JCS), and MMSE.

### Interventions

Arm 1: consecutive daily irradiation; total SRT duration, 3 days.

Arm 2: irradiation at two- to three-day intervals; total SRT duration, 8 days.

Both arms are performed once a daily with total dose of 27-30 Gy in 3 fractions (9-10 Gy per fraction). The prescription dose to the PTV is 30 Gy in 3 fractions for tumors ≤2.0 cm and 27 Gy in 3 fractions for tumors > 2.0 cm.

All patients were immobilized in a spine position with the head fixed with a shell and simulated by CT with less than 2.0-mm slice thickness. All targets and organs at risk (OAR) are contoured on the RT planning system. The CT images are fused with MRI for delineation of the target and OAR. The gross tumor volume (GTV) includes an enhancing area of the contrast agent on MRI. The clinical target volume (CTV) is defined as the same as the GTV. The GTV is expanded symmetrically by 0-2.0 mm in all dimensions to create the planning treatment volume (PTV). The margin between the GTV and PTV depends on the discretion of the medical physicist in each participating institution. OAR include the eyeball, inner ear, and the brain stem, chiasma, optic nerve, and spinal cord is expanded symmetrically by 2.0 mm in all dimensions to create the planning risk organ (PRV) if these OAR are close to the PTV. The plan is created to achieve a target coverage with 95% (D95%) of the PTV receiving 100% of each prescription dose, and to provide as homogenous of a dose distribution as possible within the target (D1% < 120% of each prescription dose, D99% > 90% of each prescription dose). The dose constraint of all OAR and PRV is the maximum dose < 20 Gy. When sufficient coverage of the PTV was not achieved without deviation from the dose constraint for the OAR and PRV, the reduction of prescription dose to the acceptable range were allowed. The patient is necessarily treated using image-guided RT such as a megavoltage- or kilovoltage-CT or ExacTrac system acquired on the RT unit immediately before irradiation.

### Quality assurance for SRT

Each participating centre is required to send the SRT information of each enrolled patient to the principal investigator. The principal investigator will check the SRT data throughout the trial period and will inform each participating centre if needed.

### Systemic therapy

Patients treated with prior systemic therapy are eligible for this study. Chemotherapy treatment are not allowed during the period of SRT, but hormonal therapy and drugs for brain edema (e.g., steroid and/or glycerol) are allowed for patients with symptom burden. The enrolled patients can receive systemic therapy after SRT.

### Follow-up evaluation, assessment of efficacy, and data collection

Patients will be seen at 1 and 3 months after SRT and every 3 months thereafter. At each visit, physical examination, KPS, JCS, toxicities, and MRI of the brain are evaluated. MMSE is evaluated every 6 months after SRT. Since poorly timed MRI may be required depending on symptom, the time of MRI at plus or minus 1 month is allowed. The adverse events will be evaluated using CTCAE ver. 5.0. Table 1 shows the follow-up schedule. After progression at an intracranial site, additional treatment (e.g., WBRT) is at the discretion of the radiation oncologists. Anonymized data will be transmitted to the principal investigator from participating centres via the case reporting form. Anonymized data will be collected before SRT (baseline) and at 1 and 3 months after SRT and every 3 months thereafter according to Table 1. When any treatment-related death, any Grade 4 toxicity, and any death during the SRT periods or within 30 days of SRT is observed, the toxicity reporting form will be transmitted to the principal investigator from participating centres.

**Table 1:** Pre- and post-treatment evaluation and assessment of efficacy

	Baseline	Treatment	Follow-up after				
Assessment^a^			1 M	3 M	6 M	Every 3 M thereafter	Every 6 M thereafter
Medical history	✓						
Physical examination	✓		✓	✓	✓	✓	
CT or PET-CT^b^	✓						
KPS	✓		✓	✓	✓	✓	
JCS	✓		✓	✓	✓	✓	
MMSE	✓				✓		✓
Brain MRI	✓		✓	✓	✓	✓	
Adverse events		✓	✓	✓	✓	✓	

Footnotes: *M* Months, *CT* Computed tomography, *PET-CT* ^18^F-fluorodeoxyglucose positron emission tomography-computed tomography, *KPS* Karnofsky performance status, *JCS* Japan Coma Scale, *MMSE* Mini mental status examination, *MRI* Magnetic resonance imaging

^a^Extra imaging outside of the study schedule is allowed per the discretion of the physician

^b^Either enhanced CT or PET-CT is required

## Statistical analysis

### Randomization

The study will employ a 1:1 randomization between Arm 1 (consecutive daily irradiation) and Arm 2 (irradiation at two- to three-day intervals), based on the stratification factors described in the Design section. Random assignment of treatment groups is centrally managed by Tatsuya Kawai using the web-based system of Mujinwari (Iruka System Ltd., Tokyo, Japan) and is balanced with randomly permuted blocks. Tatsuya Kawai is a member of data managing team and is not involved in enrollment. The other co-investigators are responsible for enrollment. Interventions will be initiated within 14 days of randomization.

### Sample size

We use a randomized phase II screening design, with a two-sided α of 0.20 and a power of 80% as recommended for our current study [12]. In a phase II screening design, the α level is set higher than the level of a phase III design (i.e., 0.05). Even if the phase II trial is positive (i.e., *p* < 0.20), that is not considered conclusive without a subsequent phase III trial. The results of the previous study of irradiation at intervals demonstrated a 1-year IC-LC of 93% [13]. Assuming a 1-year IC-LC of 70% for Arm 1 (consecutive daily irradiation) and 90% for Arm 2 (irradiation at two- to three-day intervals) and a 1-year OS of 50% for target patients, with 80% power, alpha of 0.20, an 5% lost follow-up rate, accrual time of 4 years and a total trial time of 5 years, 70 patients will be required (35 in Arm 1 and 35 in Arm 2). The secretariat of the study will contact all co-investigators to facilitate enrollment regularly.

### Primary endpoint

The intention-to-treat analyses will be performed, and patients will be analyzed in the groups to which they are assigned. The IC-LC rate will be calculated using the Kaplan-Meier method and differences will be compared using the stratified log-rank test. Only recurrence of initially treated sites will be treated as event and death will not be considered to be competing event. When enrolled patient dies, the data at the last follow up will be used to calculate the IC-LC rate. Cox proportional hazards multivariable regression analysis will be used to determine baseline factors predictive of primary endpoint.

### Secondary endpoints

The IC-PFS rate will be calculated using the Kaplan-Meier method and differences will be compared using the stratified log-rank test. Only development of initially treated sites and new BM will be treated as event and death will not be considered to be competing event. When enrolled patient dies, the data at the last follow up will be used to calculate the IC-PFS rate. The OS rate will be calculated using the Kaplan-Meier method and differences will be compared using the stratified log-rank test. OS was defined as the time from the date of randomization to death from any cause.

The rates of grade 2 or higher toxicity will be compared between groups using the Fisher’s Exact Test. The non-worsening of KPS and MMSE scores at 6- and 12-months post-enrollment will be measured with differences between groups tested using the Fisher’s Exact Test. When enrolled patient dies, the data at the last follow up will be used. For KPS, a decrease in KPS of ≥10% will be considered a decline in KPS and sensitivity analysis will be performed. For MMSE, a decrease in MMSE score of ≥3 points will be considered a decline in cognitive function and sensitivity analysis will be performed.

### Interim analysis and monitoring

Because a small sample size is required in this study, interim analysis will not be conducted. However, the safety of this trial will be independently evaluated by the data monitoring committee (DMC) when 35 and 70 patients are accrued. Principal Investigator reports about progress of the clinical trial, data correction, toxicities, and protocol deviation and violation to the DMC. The DMC is composed by the Clinical Research Center of Nagoya City University and is independent from the clinical trial team. Here is the item for the monitoring.

1. A registration status
2. A consideration of eligibility for a patient enrolled in this study
3. Any serious adverse event associated with this SRT procedure occurs
4. A violation or deviation from this study protocol
5. A critical problem for a progress, safety, and reliability of this study
6. A compliance of an ethical guideline

### Ethical considerations and institutional review board

The principal investigator obtained ethical approval and clinical trial authorization by competent authorities according to local laws and regulations. The written informed consent form provided to potential participants should be adhere to the ethical principles that have their origin in the Declaration of Helsinki and must be approved by IRB. The protocol including the case reporting form, the informed consent form, and any other written information to be given to potential participants was centrally reviewed and approved by the IRB of Nagoya City University Graduate School of Medical Sciences (approval number: 46-22-0004). All hospitals participating in this study will obtain IRB approval prior to local initiation.

### Informed consent

Informed consent to participate will be obtained from all of the participants. The investigator should obtain written informed consent from each patient before starting any study procedures and treatment. The investigator should inform the patient, or the patient’s legally acceptable representative, of the potential risks, benefits, and all aspects of the study. In addition, the investigator should inform the patient that participation in the study is completely voluntary and that they can refuse voluntarily to enter the study or to withdraw from it at any time, for any reason. The informed consent must be signed and dated by all of the patients.

### Subject discontinuation/ withdrawal

When patients discontinue participation in the study, the clinical and laboratory evaluations that would have been performed at the end of the study should be obtained. If a subject is removed because of an adverse event, they needed to be observed by the treating physician as long as deemed appropriate.

### Confidentiality

The personal information of study participants will be held in utmost secrecy. All study records will only identify the subject by initials and the study identification number assigned by data managing team. Access to proprietary information is only permitted for direct subject management and for those involved in monitoring the study. The names and personal information of study participants will not be opened in any study report. Akira Torii is a member of data managing team and has access to data of the case reporting form sent from investigators.

### Protocol amendments and trial publication

Any protocol amendments, regarding eligibility criteria, outcomes, and analyses, must be enacted by the principal investigator. Protocol amendments will be reported to all participating hospitals, investigators, IRBs, and trial registries through the principal investigator. Any conference presentation or publication of results of the study will be led by the principal investigator within 1 year after completion of trial. The results will be submitted to a peer-reviewed journal and will be presented at domestic and international conferences. Authorship of the conference presentation and scholarly paper will be decided by the principal investigator with reference to general guideline regarding qualification for authorship.

## Discussion

To date, the clinical benefit of SRT has been demonstrated for several types of cancers or metastatic lesions. In clinical practice, however, various irradiation intervals such as consecutive daily and one- to three-day intervals) are used in view of radiation biology. However, evidence to support the significance of adding a few days intervals between high-dose irradiation is unclear. SRT is widely available especially for early-stage non-small-cell lung cancer and BM due to its significant therapeutic effect. Longer follow-up time is usually necessary in lung cancer than BM to evaluate local control adequately after SRT. In addition, it is often clear in BM to evaluate local recurrence after SRT using a contrast-enhanced MRI. It is often difficult in lung cancer to assess local control after SRT because radiation pneumonitis and fibrotic changes often develop, and the tumor usually becomes indistinguishable from the fibrotic shadow. Thus, this study is designed for BM patients to assess the optimal interval of SRT.

It is well known that high oxygen levels make the tumor cells more effective to irradiation and hypoxic tumor cells are resistant to irradiation [6]. A cell population in the absence of oxygen requires a 3-fold larger radiation dose for the same amount of tumor cell kill as a cell population under physiological oxygen conditions [14]. Hypoxia is observed in many human tumor cells and can cause a recurrence of cancer after RT. Several investigators have shown clearly that the extent of tumor hypoxia has a negative impact on locally control after RT [7, 8]. However, hypoxic tumor cells reoxygenate during fractionation and get more sensitive to successive irradiation [10]. This process can be explained by fluctuating tumor blood flow [9].

**Fig. 3:**
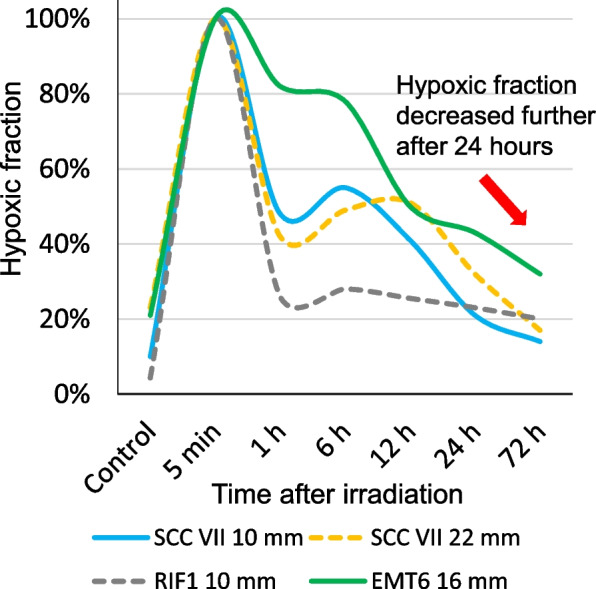
Changes in the hypoxic fraction after single high-dose irradiation in murine tumors. SCC IV, RIF1, and EMT6 shows cell line and 10 mm, 22 mm, and 16 mm shows each tumor size

Figure 3 shows the variations in the percentage of hypoxic tumor cells after single high-dose (13–15 Gy) irradiation in three types of murine tumors [11]. In all tumors, the hypoxic fractions at 1 h after irradiation were significantly lower than those immediately after irradiation. This process could be explained by reoxygenation. In all tumors, reoxygenation took more than 24 hours for the hypoxic fraction to return to the level before irradiation. In all tumors too, the hypoxic fractions tended to decrease further after 24 hours. For example, in SCC VII with 22 mm diameter, the hypoxic fraction was twice at 24 hours after irradiation as 72 hours: 32% vs 17%. When a hypoxic tumor cell requires approximately a 2 or 3-fold larger radiation dose to produce the same amount of killing tumor cells, reoxygenation is extremely important phenomenon to enhance therapeutic efficacy in SRT with hypofractionation. We think that a 24-hour interval between fractions (i.e., daily irradiation) may not be optimal in SRT with high dose per fraction, and longer intervals may be necessary to facilitate more reoxygenation and enhance therapeutic efficacy of SRT. This multicenter open-label randomized phase II trial will demonstrate an assessment of the impact of SRT interval on local control, survival, and toxicity for patients with 1–5 BM.

### Trial status

The study began in September 2022 and the trial is ongoing. Patient recruitment is expected to be completed in March 2026. Follow-up and data collection will be completed in March 2027. The final results are expected in March 2028.

## Data Availability

Not applicable.

## Abbreviations

SRT: Stereotactic radiotherapy
BM: Brain metastases
OS: Overall survival
RT: Radiotherapy
IC-LC: Intracranial local control rate
IC-PFS: Intracranial progression-free survival
KPS: Karnofsky performance status
MMSE: Mini mental status examination
MRI: Magnetic resonance imaging
WBRT: Whole brain radiation therapy
18-FDG: ^18^F-fluorodeoxyglucose
PET: Positron emission tomography
CT: Computed tomography
JCS: Japan Coma Scale
OAR: Organs at risk
GTV: Gross tumor volume
CTV: Clinical target volume
PTV: Planning treatment volume
PRV: Planning risk organ
D1%, D95%, D99%: Coverage with 1, 95, and 99% of target
